# Melatonin regulates the circadian rhythm to ameliorate postoperative sleep disorder and neurobehavioral abnormalities in aged mice

**DOI:** 10.1111/cns.14436

**Published:** 2023-09-22

**Authors:** Xixi Jia, Yanan Song, Zhengqian Li, Ning Yang, Taotao Liu, Dengyang Han, Zhuonan Sun, Chengmei Shi, Yang Zhou, Jie Shi, Yajie Liu, Xiangyang Guo

**Affiliations:** ^1^ Department of Anesthesiology Peking University Third Hospital Beijing China; ^2^ National Institute on Drug Dependence and Beijing Key Laboratory of Drug Dependence Peking University Beijing China

**Keywords:** circadian rhythm, clock genes, ERK/CREB signaling pathway, melatonin, postoperative neurobehavioral abnormalities, postoperative sleep disorders, suprachiasmatic nucleus

## Abstract

**Background:**

Postoperative sleep disorder (PSD) and delirium, which may be associated with surgery and inhalational anesthetics, induce adverse effects in old adults. Emerging evidence indicates that circadian rhythm contributes to various neuropathological diseases, including Alzheimer's disease. Thus, we analyzed the potential role of circadian rhythm in PSD and delirium‐like behavior in aged mice and determined whether exogenous melatonin could facilitate entrainment of the circadian rhythm after laparotomy under sevoflurane anesthesia.

**Methods:**

We selected old C57BL/6J mice which receiving laparotomy/sevoflurane anesthesia as model animals. We employed buried food, open field, and Y maze test to assess delirium‐like behavior, and electroencephalography/electromyography (EEG/EMG) were used to investigate sleep changes. We analyzed the transcription rhythm of clock genes in superchiasmatic nucleus (SCN) to explore the effects of surgery and melatonin pretreatment on the circadian rhythm. Then, we measured melatonin receptor levels in SCN and ERK/CREB pathway‐related proteins in hippocampus and prefrontal cortex to assess their role in PSDs and delirium‐like behavior.

**Results:**

Laparotomy under sevoflurane anesthesia had a greater influence than sevoflurane alone, leading to sleep disorder, a shift in sleep–wake rhythm, and delirium‐like behavior. Bmal1, Clock, and Cry1 mRNA expression showed a peak shift, MT_1_ melatonin receptor expression level was increased in the SCN, and p‐ERK/ERK and p‐CREB/CREB were decreased in hippocampus and prefrontal cortex of aged mice 1 day after laparotomy. Melatonin showed significant efficacy in ameliorating PSD and delirium‐like behavior and restoring the circadian rhythm, reversing melatonin receptor and ERK/CREB pathway expression abnormalities. In addition, most of the beneficial effect of melatonin was antagonized by luzindole, a melatonin receptor antagonist.

**Conclusions:**

Melatonin receptors in SCN, circadian rhythm, and ERK/CREB signaling pathway participate in the pathophysiological processes of PSD and delirium‐like behavior. Melatonin intervention could be a potential preventative approach for PSD and delirium.

## Graphical abstract

The circadian rhythm of clock genes in the SCN is analyzed to explore the effects of surgery and melatonin pretreatment on the circadian rhythm. The study is the first evaluation of the influence of melatonin receptors in the SCN, the circadian rhythm, and the ERK/CREB pathway on sleep disorders and delirium‐like behavior in aged mice.

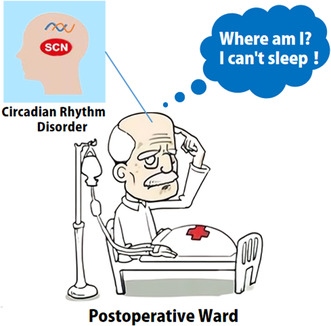

## 1 INTRODUCTION

Postoperative delirium (POD), one of the most common postoperative complications among older adults, is characterized by inattention and cognitive disturbances after surgery and is often accompanied by sleep disorders.^1^, ^2^, ^3^ It is consistently associated with prolonged hospital stays, increased mortality, and worse functional recovery.^4^ Although current studies have explored the roles of inflammation, reduced cerebral oxidative metabolism, and other factors in delirium,^5^ the neuropathogenesis of POD remains mostly unknown, and effective prevention strategies are controversial.

Postoperative sleep disorder (PSD) mainly manifests as a pathological sleep–wake rhythm disorder and change in sleep structure and phase.^6^ In total, 42% of patients report sleep disorders after surgery, and 24% of patients needing drug therapy for these disorders.^7^, ^8^ Several studies have reported that decreased cognitive functioning is associated with negative sleep changes in the elderly.^9^, ^10^ Sleep disturbance and cognitive and memory decline are common in older adults.^11^, ^12^ Animal studies have shown that sleep disorders and behavioral abnormalities may concomitantly occur in aging and Alzheimer's disease and can be associated with circadian rhythm disruption.^13^, ^14^ Increasing evidence suggests that circadian rhythm changes may influence cognitive function during aging, and sleep and diurnal interventions have been associated with a decreased incidence of POD.^15^, ^16^ These findings suggest that the circadian rhythm may play a role in neurobehavioral abnormalities in elderly. Recent studies have found that isoflurane anesthesia aggravates circadian dysrhythmia and triggers long‐term memory deficits in aged mice.^17^ However, it is still unclear how sleep changes affect neurological behavior and whether the circadian rhythm plays a role in sleep and neurobehavioral changes in aged mice after surgery.

Melatonin, which is secreted by the pineal gland, is the neuroendocrine basis of sleep–wake cycle regulation.^18^ Zhang et al.^19^ reported that exogenous melatonin tended to prolong sleep duration and reduce the prevalence of POD in patients. Our previous research showed that prophylactic melatonin can reduce anesthesia‐induced cognitive impairment in aged mice, and melatonin receptors are involved in this process.^20^ These studies suggest that melatonin may ameliorate both PSD and neurobehavioral abnormalities. Because several studies have implicated melatonin in circadian rhythm regulation through melatonin receptors^21^ and suggested that melatonin receptors may be involved in several neuropathological processes,^22^, ^23^ our study will discuss whether circadian rhythm regulation by melatonin improves PSD and delirium‐like behavior in aged mice and investigate the role of melatonin receptors in this mechanism.

Recently, two studies showed that the ERK/CREB pathway plays a role in sleep regulation and depression‐like behavior.^24^, ^25^ A study indicated that MAPK/ERK pathway oscillation in the hippocampus, which is required for memory maintenance, is driven by the suprachiasmatic nucleus (SCN, the circadian rhythm center).^26^ Thus, we tested the hypothesis that the ERK/CREB pathway, which may be regulated by the circadian rhythm, is required to induce delirium‐like behavior after surgery.

In this study, we employed a battery of tests of natural and learned behaviors to assess delirium‐like behavior,^27^, ^28^ and electroencephalography/electromyography (EEG/EMG) were used to investigate sleep changes. We analyzed the diurnal rhythm of clock genes in the SCN to explore the effects of surgery and melatonin pretreatment on the circadian rhythm. Then, we assessed the role of melatonin receptors and the ERK/CREB pathway in sleep disorders and delirium‐like behavior by measuring melatonin receptor levels in the SCN and ERK/CREB pathway‐related proteins in the hippocampus and prefrontal cortex. To our knowledge, this is the first evaluation of the influence of melatonin receptors, the circadian rhythm, and the ERK/CREB pathway on sleep disorders and delirium‐like behavior in aged mice.

## 2 MATERIALS AND METHODS

### 2.1 Animals

In total, 18‐month‐old specific pathogen‐free C57BL/6J mice (weighing 20–25 g, 50% males) were housed in an insulated and sound‐proof room under an automatically controlled 12 h/12 h light/dark cycle and given food and water ad libitum. Lights were turned on at 8:00 a.m.—Zeitgeber time (ZT) 0— and turned off at 8:00 p.m. (ZT12). All the experimental procedures met NIH guidelines for appropriate care and use of animals in research.

### 2.2 Drug administration

Intraperitoneal (i.p.) melatonin injections (Sigma Aldrich) were administered daily (10 mg/kg body weight) at ZT12 for 7 consecutive days. Melatonin was dissolved in 0.5% absolute ethanol and saline. Luzindole (1 mg/kg i.p., a competitive MT_1_/MT_2_ melatonin receptor antagonist; Tocris) was administered at ZT12 for 7 consecutive days before laparotomy under sevoflurane (Abbvie) anesthesia.

### 2.3 Experimental design

Mice that were administered the vehicle (0.5% absolute ethanol) or melatonin prior to sevoflurane anesthesia and/or laparotomy were randomly assigned to the following experiments.

#### 2.3.1 Experiment A

**FIGURE 1:**
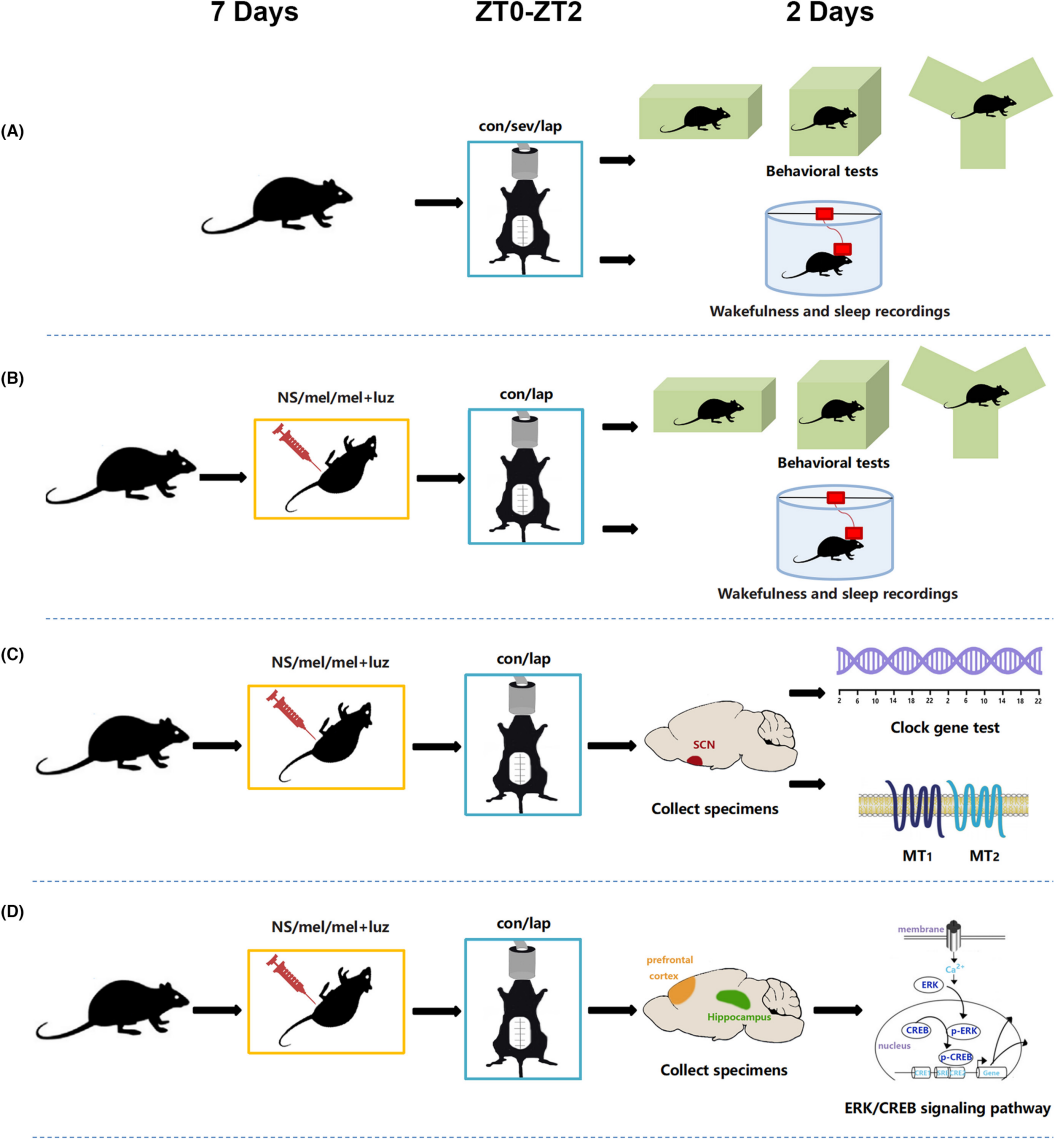
Schematic presentation of experimental design. (A) Experiment A; (B) Experiment B; (C) Experiment C; (D) Experiment D. con, control; lap, laparotomy under sevoflurane anesthesia; mel, melatonin; mel + luz, melatonin+luzindole; NS, normal saline; sev, sevoflurane anesthesia.

To investigate the effects of anesthesia and/or laparotomy on sleep and neurobehavior, mice were randomly assigned to three groups (*n* = 17 per group): control (C group), sevoflurane anesthesia (A group), and laparotomy under sevoflurane anesthesia (A/S group). C group animals were placed in their home cage with 22% oxygen for 2 h, while the other two groups were treated with sevoflurane anesthesia and laparotomy. Wakefulness and sleep recordings (*n* = 7 per group) and behavior tests (buried food, open field, and Y maze test, *n* = 10 per group) were started on days 1 and 2 (D1 and D2) post‐anesthesia/laparotomy, respectively (Figure 1A).

#### 2.3.2 Experiment B

To explore the effect of melatonin on postoperative sleep and neurobehavior, another cohort of mice was randomly exposed to melatonin + laparotomy under sevoflurane anesthesia (M + A/S group), melatonin + luzindole + laparotomy under sevoflurane anesthesia (M + L + A/S group), and melatonin alone (M group), with 17 mice in each group. Because a previous study showed that MT_1_ receptor expression was not significantly affected by luzindole administration alone, no luzindole treatment group was included.^29^ Following the pretreatment phase, mice in the M + A/S and M + L + A/S groups underwent laparotomy under sevoflurane anesthesia; mice in the M group received no treatment (Figure 1B). Wakefulness and sleep recordings and behavior tests were performed as in Experiment A.

#### 2.3.3 Experiment C

To investigate the contribution of the circadian rhythm to postoperative sleep and neurobehavior disorders and the effect of melatonin on these disorders, we investigated the transcriptional activity of clock genes (Clock, Bmal1, Cry1, and Per1) in the SCN using quantitative reverse transcription polymerase chain reaction (PCR) and melatonin receptor (MT_1_ and MT_2_) protein expression in the SCN using western blotting. Mice (*n* = 180) were randomly divided into three groups: C, A/S, and M + A/S. Tissue samples were obtained at 0, 4, 8, 12, 16, 20, 24, 28, 32, 36, 40, and 44 h (*n* = 5 at each time point) after treatment. Melatonin receptors were assayed in the C, A/S, M + A/S, M + L + A/S, and M groups at 6, 12, and 48 h (*n* = 6 at each time point), and immunofluorescence assays of melatonin receptors were performed 6 h after treatments (Figure 1C).

#### 2.3.4 Experiment D

We aimed to determine whether circadian rhythm correction improved postoperative neurobehavioral abnormalities via MAPK/ERK signaling pathway activation. ERK and CREB expression and phosphorylation were assayed at 12, 24, and 48 h following treatments by western blots (five groups, similar to Experiment C, *n* = 6 at each time point) and immunofluorescence (Figure 1D).

### 2.4 Sevoflurane exposure and exploratory laparotomy

Sevoflurane exposure was performed according to a previously described protocol.^30^ Briefly, sevoflurane exposure and/or laparotomy was started at ZT0. Mice were exposed to 2% sevoflurane in 22% oxygen for 2 h in an anesthesia chamber; the concentration was monitored with a gas outlet. The heart rate, blood‐oxygen saturation, and rectal temperature were monitored. The mice breathed spontaneously, and sevoflurane was well tolerated, with all monitored variables in the physiological range.

Laparotomy was aseptically performed with a method previously used in mice.^31^ Mice were anesthetized for 2 h with 2% sevoflurane and intracutaneously injected with 0.2% ropivacaine along the planned incision line. A 2‐cm vertical incision was made in the middle of the abdomen, the gastrointestinal tract was exteriorized and vigorously rubbed for 30 s, and the organs (liver, spleen, kidneys, and bowel) were gently probed with cotton for 30 min. The intestines were then placed back into the peritoneal cavity, and the skin was sutured with surgical staples. EMLA cream (2.5% lidocaine and 2.5% prilocaine, AstraZeneca, Sweden) was applied to the incision wound at the end of surgery and then every 8 h for 2 days for surgical pain relief. Body temperature was maintained with a heating pad during anesthesia/surgery.

### 2.5 Behavior tests

A battery of behavioral tests described in a previous study was performed to assess changes in both natural and learned behaviors following surgery and anesthesia to examine POD.^32^ All the mice underwent the tests in the following order: buried food, open field, and Y maze test at 24 h before anesthesia/surgery (baseline) and at 6, 9, 24, and 48 h after anesthesia/surgery. The behavior tests were completed within 50 min to simulate clinical evaluation of delirium in patients. The latency to eating food (buried food test); time spent in the center, latency to the center, and total distance traveled (open field test); and duration and entries in the novel arm and total distance traveled (Y maze test) were recorded. All the behavioral data were analyzed with an animal tracking system (Smart 3.0, RWD Life Science Co., Ltd).

### 2.6 Polygraphic recordings and vigilance state analysis

Polygraphic recordings and vigilance state analysis were performed according to a previous study.^33^ In brief, mice were implanted with electrodes for polysomnographic EEG and EMG recordings under 2% sevoflurane anesthesia. The implant comprised two stainless‐steel screws (1 mm diameter) serving as EEG electrodes, one of which was placed epidurally over the right frontal cortex (1.0 mm anterior and 1.5 mm lateral to bregma) and the other over the right parietal cortex (1.0 mm anterior and 1.5 mm lateral to lambda). Two insulated Teflon‐coated, silver wires (0.2 mm in diameter), which were placed bilaterally into the trapezius muscles, served as EMG electrodes. All the electrodes were attached to a micro‐connector and fixed onto the skull with dental cement. The mice were given intracutaneous injections of 0.2% ropivacaine along the wounds once per day for 2 days. After the surgical procedure, mice were maintained undisturbed in the housing room for 14 days. The mice were habituated to the recording cable for 2 days before polysomnographic recording. Baseline recordings of wakefulness and sleep were conducted for 2 days prior to treatment. Wakefulness and sleep recordings were started immediately after 2 h of anesthesia/laparotomy and lasts for 2 days.

The EEG/EMG signals were amplified and filtered (EEG, 0.5–30 Hz; EMG, 20–200 Hz), digitized at a sampling rate of 128 Hz, and recorded using SLEEPSIGN software (KISSEI COMTEC CO., LTD.). The vigilance states were automatically classified offline, in 4‐s epochs, into rapid eye movement (REM) sleep, nonrem (NREM) sleep, and wakefulness by SLEEPSIGN according to the standard criteria.^34^ As a final step, defined sleep–wake stages were examined visually and corrected if necessary.

### 2.7 Western blot analysis

Western blotting was performed to determine the CREB, phosphorylated (p)‐CREB, ERK, and p‐ERK protein levels in the hippocampus and prefrontal cortex and the MT_1_ and MT_2_ receptor levels in the SCN. Briefly, the primary antibodies used for western blotting were as follows: anti‐MT_1_ receptor antibody (1:1000; Abcam, Cambridge, UK, ab203038), anti‐MT_2_ receptor antibody (1:100; Abcam, ab203346), anti‐ERK1/2 antibody (1:200; Abcam, ab17942), anti‐p‐ERK1/2 antibody (1:50; Santa Cruz Biotechnology, sc‐81492), anti‐CREB antibody (1:1000; Abcam, ab32515), anti‐p‐CREB antibody (1:1000; Abcam, ab32096), and β‐actin (1:5000; Servicebio, GB12001). Band intensities were quantified by infrared scanning densitometry (Odyssey Imaging Systems; LI‐COR Biosciences).

### 2.8 Immunofluorescent staining

Immunofluorescence was performed to determine the p‐CREB, ERK1/2, and p‐ERK1/2 expression and distribution in the hippocampus. Briefly, the primary antibodies included p‐CREB (1:100; Abcam, ab32096), ERK1/2 (1:200; Abcam, ab17942), and anti‐p‐ERK1/2 (1:50; Santa Cruz Biotechnology, sc‐81492), and the secondary antibodies included Goat anti‐Rabbit Alexa‐Fluor 488‐ (1:200; Abcam, ab150077) and Goat anti‐Mouse Alexa‐fluor 594‐conjugated antibodies (1:200; Abcam, ab150116). Nuclei were counterstained with DAPI (1:5000; Roche, 236276). Images were captured with a confocal fluorescence microscope (Nikon DS‐U3) for analysis of the hippocampal CA3 region.

### 2.9 mRNA expression of clock genes

Total RNA was extracted from the SCN using an Eastep Universal RNA Extraction Kit (Promega) according to the standard protocol. The RNA concentrations were determined using a Nanodrop spectrophotometer (Thermo Scientific). Total RNA was reverse‐transcribed using the GoScript Reverse Transcription System (Promega). The cDNA solution was subjected to quantitative PCR in a Bio‐Rad iCycler iQ system using GoTaq® qPCR Master Mix (Promega). Quantitative PCR consisted of 40 cycles of 15 s at 95°C and 60 s at 60°C. The primer sequences were as follows:

Per1 forward primer 5′‐CTCTTCTGGCAATGGCAAGGACTC‐3′,

reverse primer 5′‐CTCAGGAGGCTGTAGGCAATGGA‐3′;

Clock forward primer 5′‐GACGGCGAGAACTTGGCATTGA‐3′,

reverse primer 5′‐TGAGACTGCGGTGTGAGATGACT‐3′;

Bmal1 forward primer 5′‐ATAAGGACTTCGCCTCTACCTGTTCA‐3′,

reverse primer 5′‐CCTCGTTGTCTGGCTCATTGTCTT‐3′;

Cry1 forward primer 5′‐GCCAGCAGACACCATCACATCAG‐3′,

reverse primer 5′‐GGGAAGGAACGCCATATTTCTCATCA‐3′;

GAPDH forward primer 5′‐AGAAGGTGGTGAAGCAGGCATCT‐3′,

reverse primer 5′‐CGGCATCGAAGGTGGAAGAGTG‐3′.

Temperature controlled melting curve analysis revealed a single peak corresponding to the specific amplification product. mRNA expression levels of clock genes in the SCN which related to the circadian rhythm, were calculated using the 2^−ΔΔCT^ method and analyzed using cosine software (Chronos‐Fit program).

### 2.10 Statistical analyses

The Shapiro–Wilk test were used to assess data distribution. Parametric results in normal distribution are presented as mean ± SEM. Statistical analyses were performed with SPSS 25.0 (SPSS Inc.). The statistical chart is drawn with GraphPad Prism 8.0. Differences were considered statistically significant at *p* < 0.05.

## 3 RESULTS

### 3.1 Laparotomy under sevoflurane anesthesia had a more significant effect on sleep and neurobehavioral disturbances in aged mice than sevoflurane anesthesia alone

**FIGURE 2:**
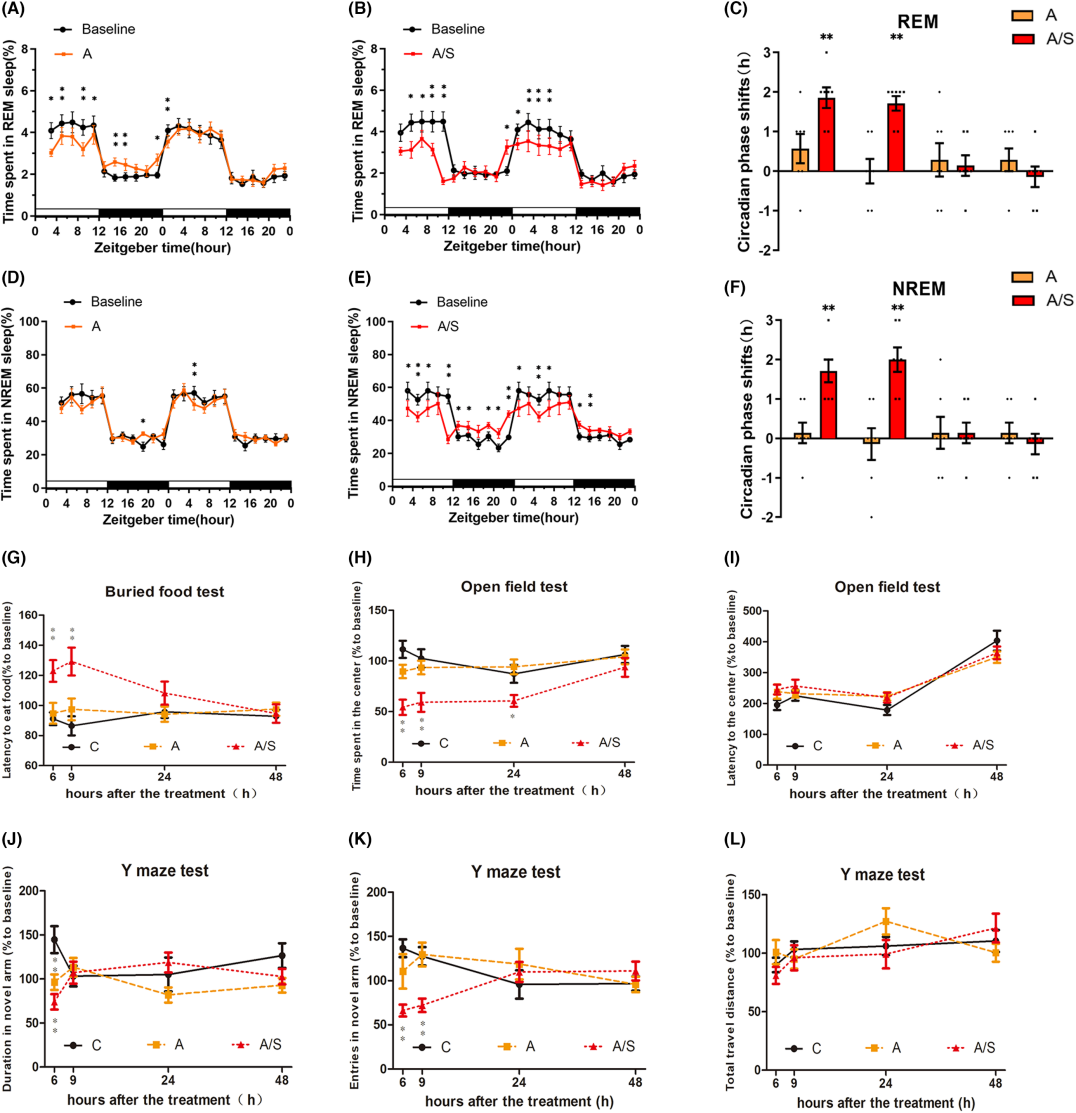
Laparotomy under sevoflurane anesthesia impaired the sleep–wake rhythm and behavior of aged mice 1 day postoperatively. (A, B, D, E) Percentages of time spent in (A, B) REM sleep and (D, E) NREM sleep each 2 h as a function of time at baseline (black) and following anesthesia (orange)/laparotomy (red). *p < 0.05 and **p < 0.01 indicate significant differences compared with their own controls, data are assessed by two‐tailed paired Student's *t*‐tests. (C, F) The circadian phase shifts in (C) REM sleep and (F) NREM sleep 2 days after anesthesia (orange)/laparotomy (red) *n* = 7. The inflection point is defined as the percentage of REM or NREM sleep in 6 periods (2 h/period) after the period at which the point is located is greater than or less than 30% of the period at which the point is located. L_1_ → D_1_: Phase transition from the first light phase to the first dark phase after anesthesia/laparotomy; D_1_ → L_2_: Phase transition from the first dark phase to the second light phase after anesthesia/laparotomy; L_2_ → D_2_: Phase transition from the second light phase to the second dark phase after anesthesia/laparotomy; D_2_ → L_3_: Phase transition from the second dark phase to the third light phase after anesthesia/laparotomy. (G–L) Impact at 6, 9, 24, and 48 h of anesthesia/laparotomy on mouse behaviors assessed by (G) the buried food test (H, I) the open field test (J–L) the Y maze test *n* = 10. Data were analyzed by repeated measures ANOVA (the different group were the between groups factors and time was the repeated measures factor), followed by a post hoc Bonferroni's multiple comparisons test. Data are presented as the mean ± SEM.

We analyzed the changes in time spent in REM and NREM sleep every 2 h after 2% sevoflurane anesthesia and laparotomy. The time course of changes revealed that the REM sleep percentage was decreased at ZT2–ZT4, ZT4–ZT6, ZT8–ZT10, ZT10–ZT12, and ZT0–ZT2(D2) after sevoflurane anesthesia and increased at ZT14–ZT16, ZT16–ZT18, and ZT22–ZT0 in mice (all *p* < 0.05, Figure 2A). The A/S group showed a decreased REM sleep percentage at ZT4–ZT6, ZT6–ZT8, ZT8–ZT10, and ZT10–ZT12 on D1 and ZT0–ZT2, ZT2–ZT4, ZT4–ZT6, and ZT6–ZT8 on D2 and an increase in the REM percentage at ZT22–ZT0 after laparotomy (all *p* < 0.05, Figure 2B).

As shown in Figure 2D, time course changes revealed that the NREM sleep percentage in mice increased at ZT18–ZT20 after sevoflurane anesthesia and decreased at ZT4–ZT6 on D2 (all *p* < 0.05). The A/S group showed a decreased NREM sleep percentage at ZT2–ZT4, ZT4–ZT6, ZT6–ZT8, ZT10–ZT12(D1) and ZT0–ZT2, ZT4–ZT6, and ZT6–ZT8(D2) and an increased percentage at ZT12–ZT14, ZT14–ZT16, ZT18–ZT20, ZT20–ZT22, and ZT22–ZT0(D1) and ZT12–ZT14 and ZT14–ZT16(D2) (all *p* < 0.05, Figure 2E).

It is noteworthy that during the ZT10–12 period, the decrease in REM and NREM sleep was greater than that in the preceding periods in the A/S group. Figure 2C,F show the inflection points of the percentages of time spent in REM and NREM sleep for each mouse. The inflection points of time spent in REM and NREM sleep were both shifted forward by approximately 2 h following the transition from the light period to the dark period (L_1_ → D_1_) and the dark period to the light period (D_1_ → L_2_) after laparotomy, but the shift disappeared on D2.

To assess the effects of anesthesia/laparotomy on the natural habits of mice, we first performed the buried food test (Figure 2G). Compared with the C group, the latency to finding food in the A/S group was longer at both 6 h (91.12 ± 4.26% vs. 122.93 ± 7.22%) and 9 h (86.48 ± 6.32% vs. 129.21 ± 9.25%) (all *p* < 0.05) but not at 24 or 48 h.

The open field test was used to evaluate whether anesthesia/laparotomy affected the fear and novelty seeking state of mice (Figure 2H,I). Laparotomy decreased the time spent in the center region at 6, 9 and 24 h (111.60 ± 8.50% vs. 54.28 ± 7.57%, 102.54 ± 8.98% vs. 59.22 ± 9.36%, 87.35 ± 8.89% vs. 60.61 ± 5.83%, all *p* < 0.05). Therefore, laparotomy under anesthesia had a time‐dependent adverse effect on the emotional state of mice.

The spontaneous Y‐maze test was performed to evaluate spatial learning and memory in the mice. Compared with the C group, both the A and A/S groups showed decreased time spent in the novel arm at 6 h (144.68 ± 15.20% vs. 96.43 ± 8.87%, 144.68 ± 15.20% vs. 74.01 ± 8.79%, all *p* < 0.05, Figure 2J). Furthermore, the A/S group showed significantly fewer entries into the novel arm than the C group at both 6 and 9 h (136.71 ± 10.09% vs. 66.10 ± 6.74%, 127.53 ± 10.45% vs. 72.05 ± 7.71%, all *p* < 0.05, Figure 2K). In addition, the total distance traveled was not significantly different at any time point (Figure 2L). The above results demonstrate that laparotomy undermines the natural ability of mice to find food, has adverse effect on the emotional state and impairs spatial learning and memory in a time‐dependent and motor‐independent manner.

### 3.2 Melatonin ameliorated sleep disorders and neurobehavioral changes induced by laparotomy under sevoflurane anesthesia, and melatonin receptor antagonists could block this effect

**FIGURE 3:**
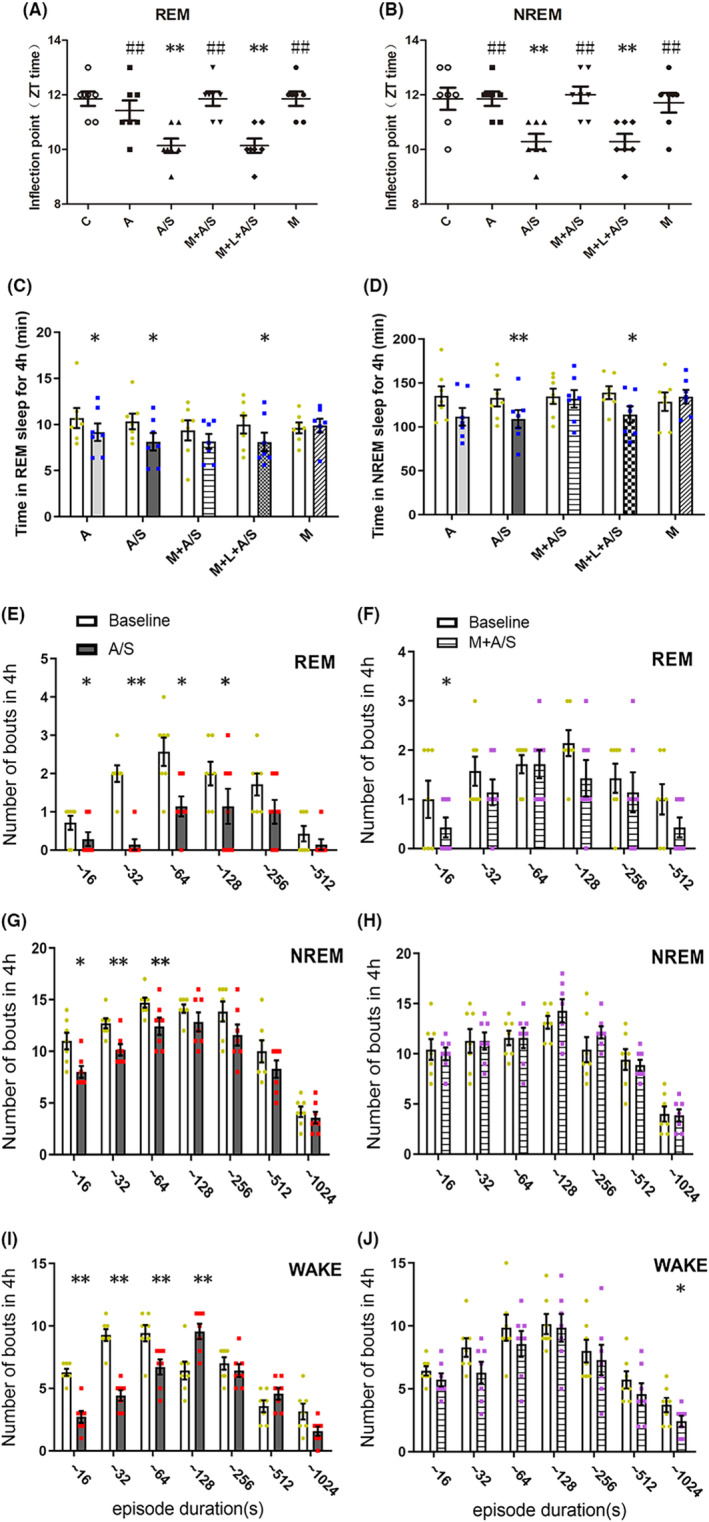
Melatonin pretreatment improved the sleep disorder caused by laparotomy under anesthesia, and melatonin antagonist can partially antagonize this improvement. (A, B) The first inflection points of time in REM sleep and NREM sleep after different treatment. Zeitgeber time (ZT) time stands for ZT. *, **p < 0.05 or 0.01 compared with C group; ^#, ##^ *p* < 0.05, 0.01 compared with A/S group. Data were analyzed by one‐way ANOVA followed by Bonferroni post hoc analysis. (C, D) Total time spent in REM sleep, NREM sleep at baseline and following treatment for 4 h light phase (ZT4–ZT8, the first day postoperatively). (E–J) Changes in the numbers of REM, NREM and wakefulness bouts across different ranges of episode durations over the course of 4 h (ZT4–ZT8, the first day postoperatively) after the different treatment. (C–J) *p < 0.05 and **p < 0.01 indicate significant differences compared with their own controls, as assessed by two‐tailed paired Student's *t*‐tests. Values are mean ± SEM (*n* = 7).

Upon further analysis of the REM and NREM sleep inflection points for all mouse groups (Figure 3A,B), we found that melatonin pretreatment reversed the forward shift of the inflection point, however, no shift was found in the M group. Because the inflection points for REM and NREM sleep were also shifted forward by approximately 2 h on D1 after laparotomy in the M + L + A/S group, we speculate that laparotomy under anesthesia can cause circadian fluctuations and melatonin may reverse these changes through melatonin receptors.

We then investigated the total time spent in REM and NREM sleep of each group from ZT4 to ZT8. The total time spent in REM sleep decreased 14.3%, 23.9%, and 18.1% in the A, A/S, and M + L + A/S groups, respectively (all *p* < 0.05, Figure 3C), which was consistent with the decreases of 18.2% and 17.8% in NREM sleep in the A/S and M + L + A/S groups, respectively (all *p* < 0.05, Figure 3D). Meanwhile, the times spent in REM and NREM sleep were unaltered in the M group. These results suggest that laparotomy under sevoflurane anesthesia had a greater impact on sleep times than did sevoflurane alone from ZT4 to ZT8, and pretreatment with melatonin can normalize the sleep disturbance.

To better understand the postoperative changes in sleep architecture, the distribution of the number of periods in each stage from ZT4 to ZT8 was determined as a function of duration (Figure 3E,G,I). Laparotomy under sevoflurane anesthesia decreased the number of periods of REM sleep with durations of 0–16, 16–32, 32–64, and 64–128 s (all *p* < 0.05). Simultaneously, the numbers of periods of NREM sleep with durations of 0–16, 16–32, and 32–64 s (all *p* < 0.05) were significantly decreased. The number of periods of wakefulness with durations of 0–16, 16–32, and 32–64 s (all *p* < 0.05) were decreased; only the number of periods of wakefulness with a duration of 64–128 s increased (*p* < 0.05). After melatonin pretreatment, only the number of periods of REM with durations of 0–16 s and wakefulness with durations of 512–1024 s were decreased (all *p* < 0.05, Figure 3F,H,J). These results suggest that the sleep architecture changed during ZT4–ZT8 after the surgery, and this change was alleviated by melatonin pretreatment.

**FIGURE 4:**
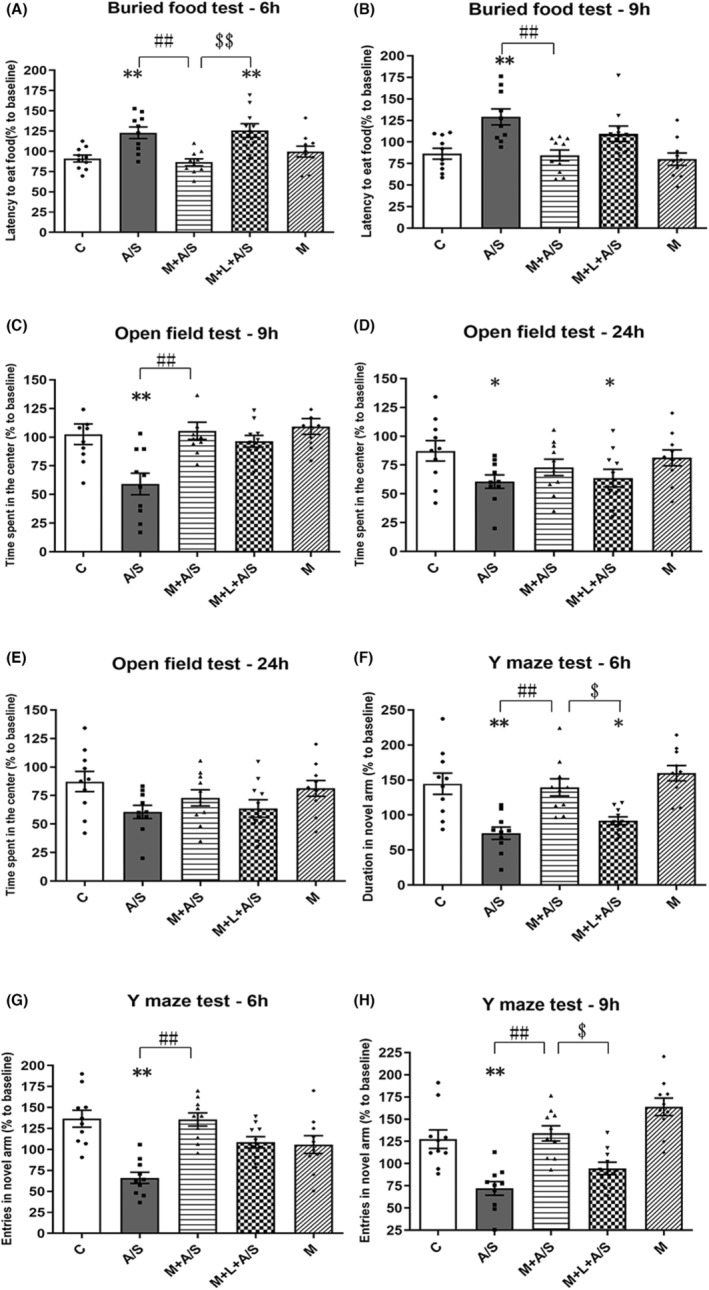
Melatonin pretreatment improved the behavior disorder caused by laparotomy under anesthesia, and melatonin receptor antagonist can partially antagonize this improvement. (A, B) the buried food test, (C–E) the open field test, (F–H) the Y maze test. *n* = 10. *p < 0.05 and **p < 0.01, compared to C group; ^#^ *p* < 0.05 and ^##^ *p* < 0.01, M + A/S group compared to A/S group; ^$^ *p* < 0.05 and ^$$^ *p* < 0.01, M + L + A/S group compared to M + A/S group; Data are presented as the mean ± SEM and analyzed by one‐way ANOVA followed by the Bonferroni post hoc analysis.

Compared with the A/S group, the latency to finding food in the M + A/S group was shorter at both 6 and 9 h (122.93 ± 7.22% vs. 86.53 ± 4.29%, 129.21 ± 9.25% vs. 84.50 ± 6.33%, all *p* < 0.05, Figure 4A,B) in the buried food test. In addition, compared with the M + A/S group, the latency to finding food in the M + L + A/S group was longer at 6 h (86.53 ± 4.29% vs. 125.67 ± 8.35%, *p* < 0.05).

In the open field test, compared with the A/S group, M + A/S group increased the time spent in the center region at 9 h (59.22 ± 9.36% vs. 105.54 ± 7.48%, *p* < 0.05) but not at 24 h (Figure 4C,D). Luzindole pretreatment did not significantly antagonize melatonin pretreatment at either 9 or 24 h. Furthermore, the total distance traveled was not significantly different among the five groups at any time point (Figure 4E), suggesting that different treatments did not cause motor dysfunction.

Compared with the A/S group, M + A/S group increased the time spent in the novel arm at 6 h (74.01 ± 8.79% vs. 139.42 ± 12.29%) in the Y maze test (Figure 4F) and increased entries into the novel arm at both 6 h (66.10 ± 6.74% vs. 135.75 ± 7.80%, Figure 4G) and 9 h (72.05 ± 7.71% vs. 134.05 ± 8.57%, all *p* < 0.05, Figure 4H). Furthermore, luzindole pretreatment showed significant antagonism in the M + L + A/S group, as the time spent in the novel arm decreased (139.42 ± 12.29% vs. 91.76 ± 5.59%) at 6 h, and the entries into the novel arm decreased (134.05 ± 8.57% vs. 94.54 ± 7.02%) at 9 h (all *p* < 0.05). These results demonstrate that melatonin can alleviate spatial learning and memory impairments caused by laparotomy under anesthesia, and melatonin receptor antagonists can partially prevent this improvement.

### 3.3 Melatonin receptor and clock gene expression in the SCN was disturbed by laparotomy under sevoflurane anesthesia, and melatonin could improve these effects

**FIGURE 5:**
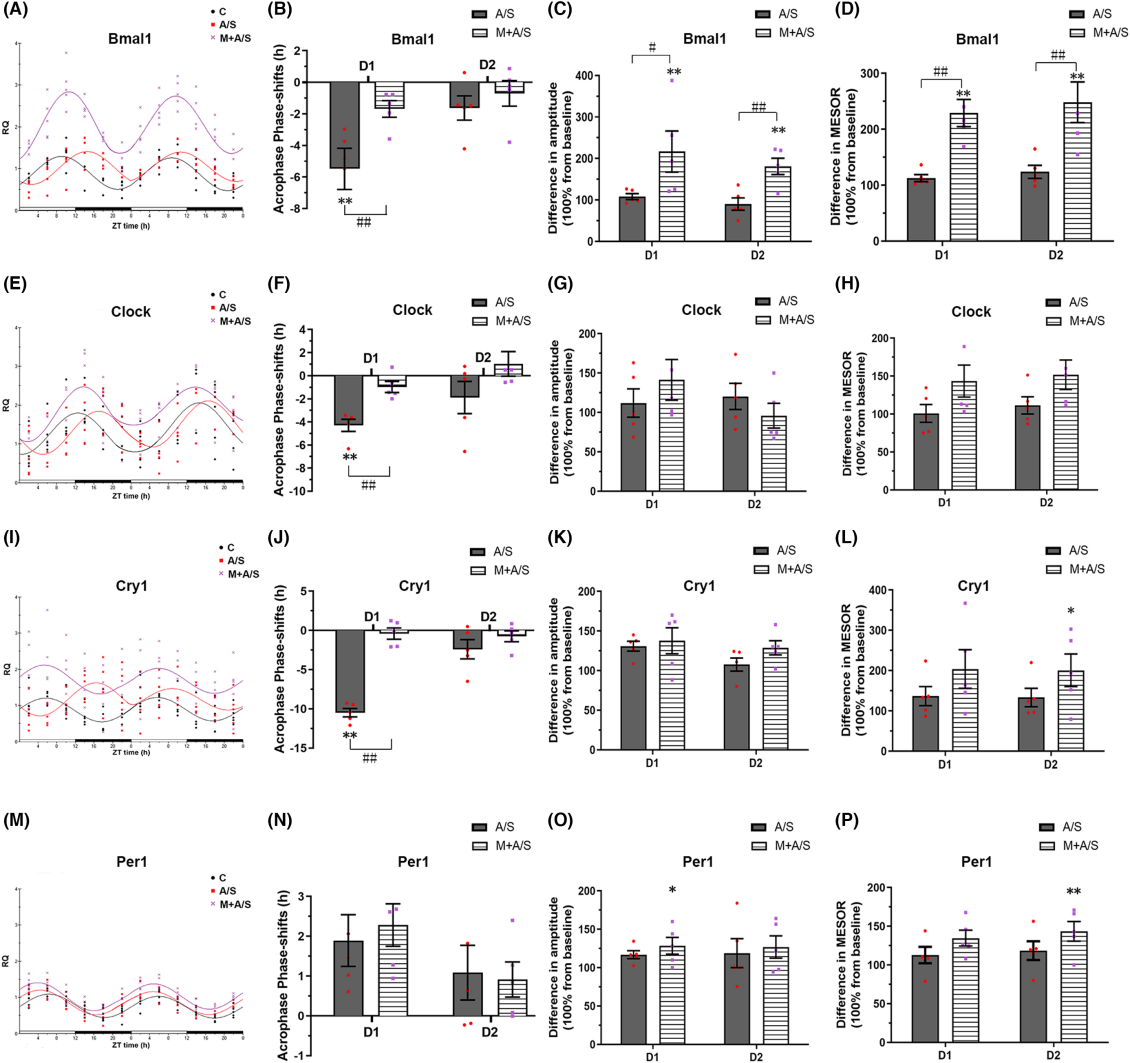
Laparotomy under sevoflurane anesthesia changed the circadian rhythm of clock gene expression in the SCN and melatonin pretreatment ameliorate the change. (A, E, I, M) Clock gene expression; The gray bar indicates the light period ZT0–ZT12, and the black bar indicates the dark period ZT12–ZT24. Black line: C group; red line: A/S group; purple line: M + A/S group. (B, F, J, N) Phase shift of Acrophase; Positive values represent phase advance. (C, G, K, O) Amplitude; (D, H, L, P) MESOR. (A, B, C, D) Bmal1; (E, F, G, H) Clock; (I, J, K, L) Cry1; (M, N, O, P) Per1. D1: The first day after laparotomy; D2: The second day after laparotomy. Results were presented as mean ± SEM (except for A, E, I, M), *n* = 5, the data were calculated by Chronos‐Fit cosine analysis software. *p < 0.05 and **p < 0.01, compared to C group; ^#^ *p* < 0.05 and ^##^ *p* < 0.01, compared to A/S group. Data were analyzed by one‐way ANOVA followed by Bonferroni post hoc analysis.

A 2 h laparotomy under sevoflurane anesthesia significantly altered clock gene expression in the SCN for 2 days after surgery, especially on D1 (Figure 5A,E,I,M). Compared with the C group, Bmal1, Clock, and Cry1 mRNA expression showed a delayed peak on D1 after surgery (5.48 ± 1.30, 4.30 ± 0.52, 10.49 ± 0.53 h, all *p* < 0.05), with no phase shift on D2. Per1 expression showed no phase shift within 2 days after surgery. It is noteworthy that melatonin restored clock mRNA expression to baseline levels after surgery (Figure 5B,F,J,N).

Laparotomy did not change the amplitude of clock gene expression; however, a change was observed in the M + A/S group (Figure 5C,G,K,O). Melatonin pretreatment increased the amplitude of Bmal1 mRNA expression in mice on D1 and D2 compared with the C group (216.71 ± 49.50% and 180.96 ± 19.67% of baseline, all *p* < 0.05). Melatonin also increased the amplitude of Per1 mRNA expression on D1 (128.45 ± 10.93% of baseline, *p* < 0.05).

Similar to the change in amplitude, melatonin pretreatment affected the MESOR of clock gene expression, but laparotomy did not cause a significant change in the MESOR compared with the C group (Figure 5D,H,L,P). Melatonin pretreatment increased the MESOR of Bmal1 mRNA expression in mice on D1 and D2 compared with the C group (229.09 ± 24.42% and 248.18 ± 36.42% of baseline, all *p* < 0.05). Melatonin also increased the MESOR of Cry1 and Per1 mRNA expression on D2 (200.61 ± 40.60% and 143.36 ± 12.66% of baseline, all *p* < 0.05).

**FIGURE 6:**
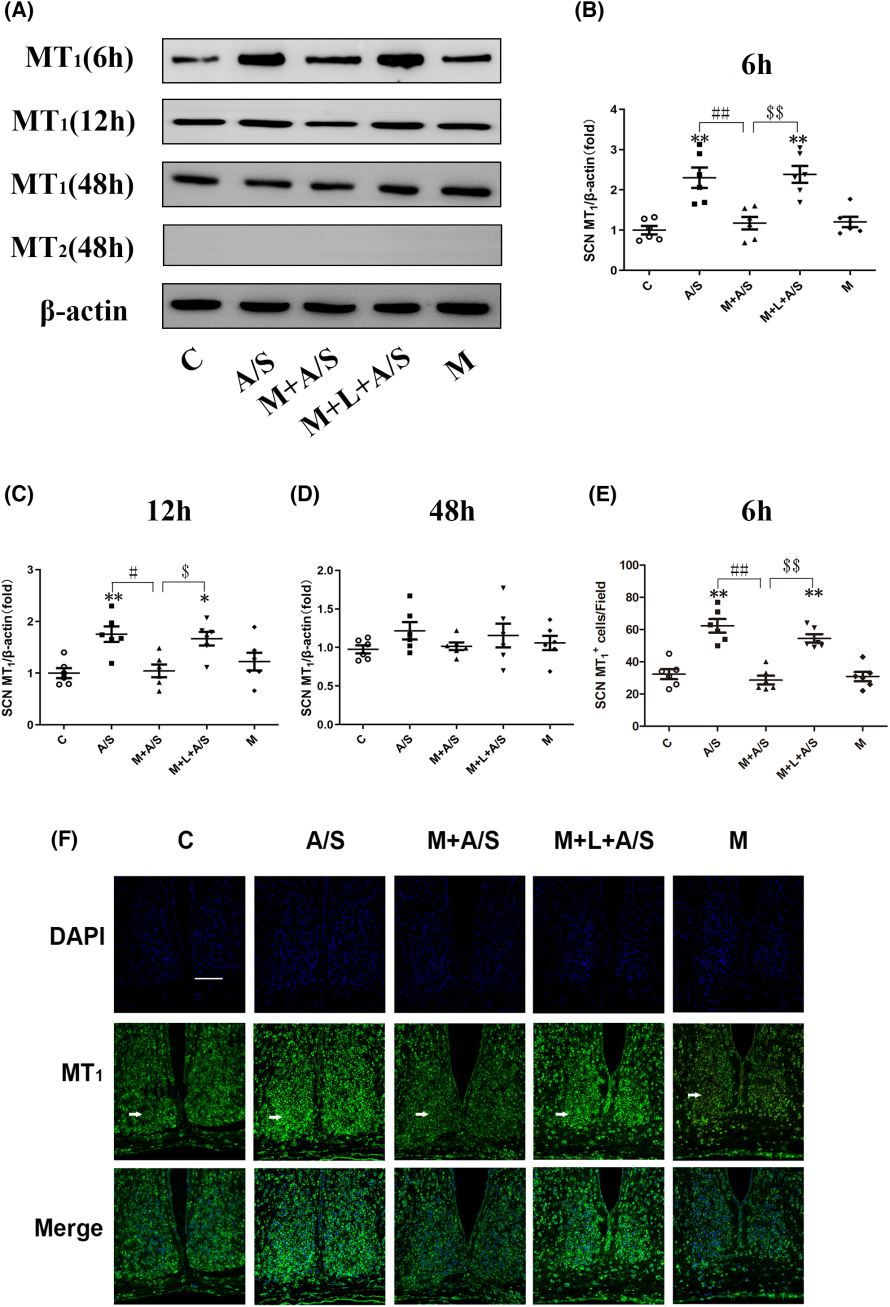
Western blotting analysis (A–D) and immunofluorescence (E, F) showed that melatonin pretreatment attenuated the increase in the expression of MT_1_ proteins in SCN induced by laparotomy at 1 day postoperatively, and the corrective action of melatonin was antagonized by luzindole. The expression of MT_2_ proteins was not detected by Western blot within 2 days after different treatment *n* = 6. *p < 0.05 and **p < 0.01, compared to C group; ^#^ *p* < 0.05 and ^##^ *p* < 0.01, M + A/S group compared to A/S group; ^$^ *p* < 0.05 and ^$$^ *p* < 0.01, M + L + A/S group compared to M + A/S group; Data are presented as the mean ± SEM and analyzed by one‐way ANOVA followed by the Bonferroni post hoc analysis.

To assess the effect of laparotomy under anesthesia on melatonin receptors in the SCN of aged mice, MT_1_ and MT_2_ receptor expression levels were analyzed by western blotting analysis and showed that laparotomy under anesthesia significantly increased MT_1_ receptor expression levels at 6 and 12 h (all *p* < 0.05) but not at 2 days after laparotomy. Pretreatment with melatonin could normalize the laparotomy‐induced changes in MT_1_ receptor levels in the SCN, and the corrective action of melatonin was antagonized by luzindole (both 6 and 12 h; all *p* < 0.05). MT_2_ receptor expression was not detected by western blots within 2 days after treatments, which suggests that MT_2_ receptor labeling was sparse in the SCN (Figure 6A–D).

We then detected MT_1_ expression in the SCN 6 h after treatment by immunofluorescence (Figure 6E,F). The results also suggested that laparotomy under anesthesia increased MT_1_ receptor expression levels in the SCN, melatonin can normalize the laparotomy‐induced change in MT_1_ receptor levels, and the corrective action of melatonin could be antagonized by luzindole (all *p* < 0.05).

### 3.4 ERK/CREB signaling pathway‐related protein expression in the hippocampus and prefrontal cortex was disturbed by laparotomy under sevoflurane anesthesia, and melatonin could ameliorate these effects

We next assessed whether the ERK/CREB signaling pathway participates in postoperative depression‐like behavior.

**FIGURE 7:**
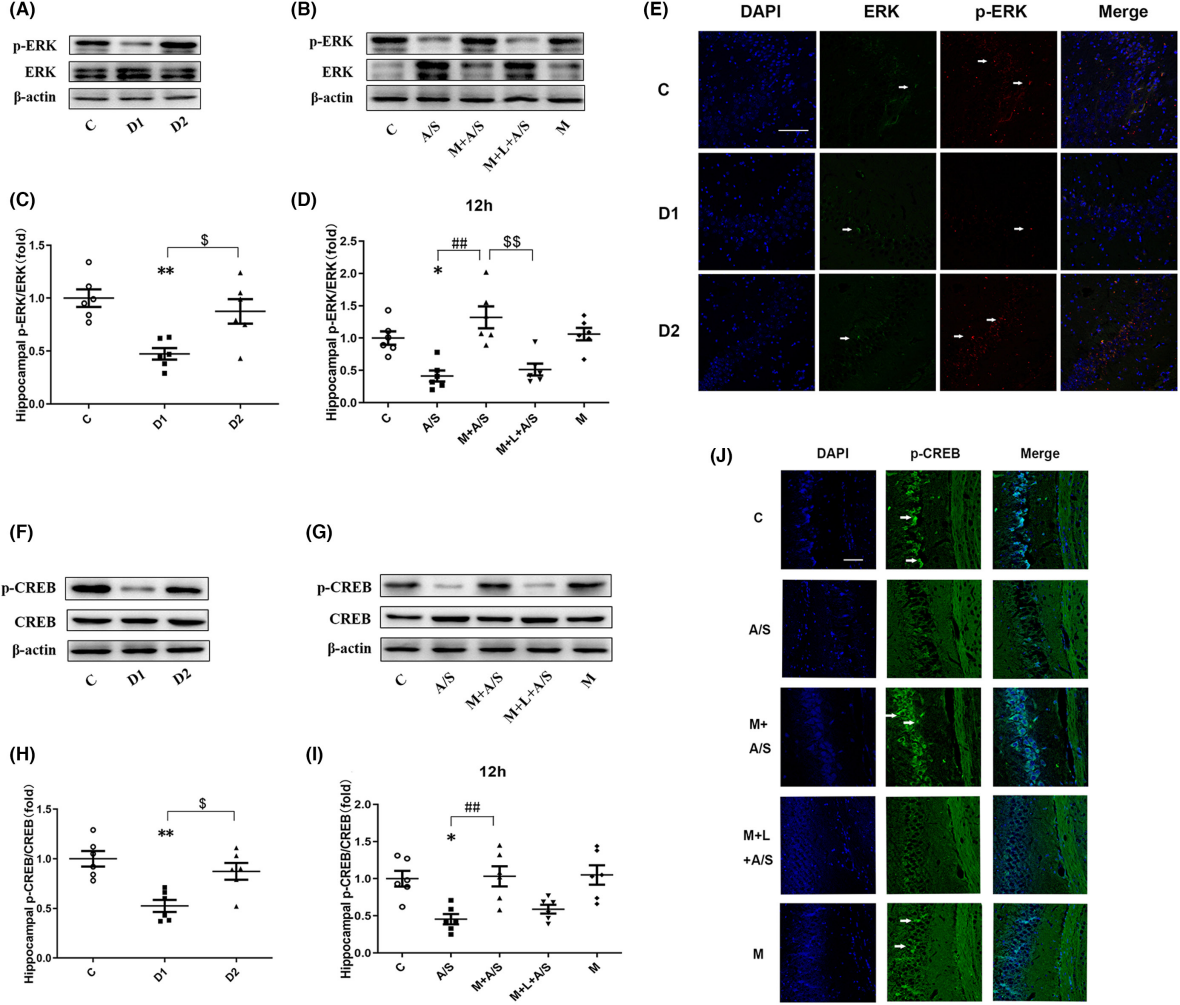
The ERK/CREB signal pathway in hippocampus can be disturbed by laparotomy and preoperative administration of melatonin can improve the expression of ERK/CREB signal pathway related proteins. (A, C, E) The Western blot analysis and Immunofluorescence showed that the expression of p‐ERK/ERK was decreased 1 day after laparotomy (D1) and returned to normal on the second day (D2). (B, D) Western blot analysis showed that melatonin pretreatment attenuated the decrease of p‐ERK/ERK expression after laparotomy and luzindole reverse the corrective action of melatonin. Melatonin alone did not change the p‐ERK/ERK expression. (F, H) Western blot analysis showed that the expression of p‐CREB/CREB was decreased 1 day after laparotomy (D1) and returned to normal on the second day (D2). (G, I, J) The Western blot analysis and Immunofluorescence showed that melatonin pretreatment attenuated the decrease of p‐CREB/CREB expression 12 h after laparotomy. Melatonin alone did not change the p‐CREB/CREB expression. D1: The first day after laparotomy in A/S group; D2: The second day after laparotomy in A/S group. *p < 0.05 and **p < 0.01, A/S group compared to C group; ^#^ *p* < 0.05 and ^##^ *p* < 0.01, M + A/S group compared to A/S group; ^$^ *p* < 0.05 and ^$$^ *p* < 0.01, M + L + A/S group compared to M + A/S group or the second day in A/S group compared to the first day after laparotomy; Data are presented as the mean ± SEM and analyzed by one‐way ANOVA followed by the Bonferroni post hoc analysis.

In the hippocampus of aged mice, p‐ERK/ERK decreased on D1 after laparotomy (*p* < 0.05) compared with the control condition and returned to normal on D2 (Figure 7A,C,E). When melatonin pretreatment was applied, the decrease in p‐ERK/ERK 12 h after laparotomy was alleviated, however, this improvement was antagonized by luzindole (all *p* < 0.05, Figure 7B,D). Similar trends were observed for p‐CREB/CREB, which were decreased on D1 after laparotomy (*p* < 0.05) and returned to normal on D2 (Figure 7F,H). When melatonin pretreatment was applied, the decrease in p‐CREB/CREB 12 h after laparotomy was alleviated (all *p* < 0.05). In contrast to p‐ERK/ERK, the decrease in p‐CREB/CREB in the M + L + A/S group was not statistically significant compared with that in the M + A/S group (Figure 7G,I,J), which may indicate that luzindole (1 mg/kg) has an insufficient antagonistic effect on p‐CREB/CREB in the hippocampus 12 h after laparotomy. In addition, melatonin alone did not trigger ERK and CREB phosphorylation in the hippocampus.

**FIGURE 8:**
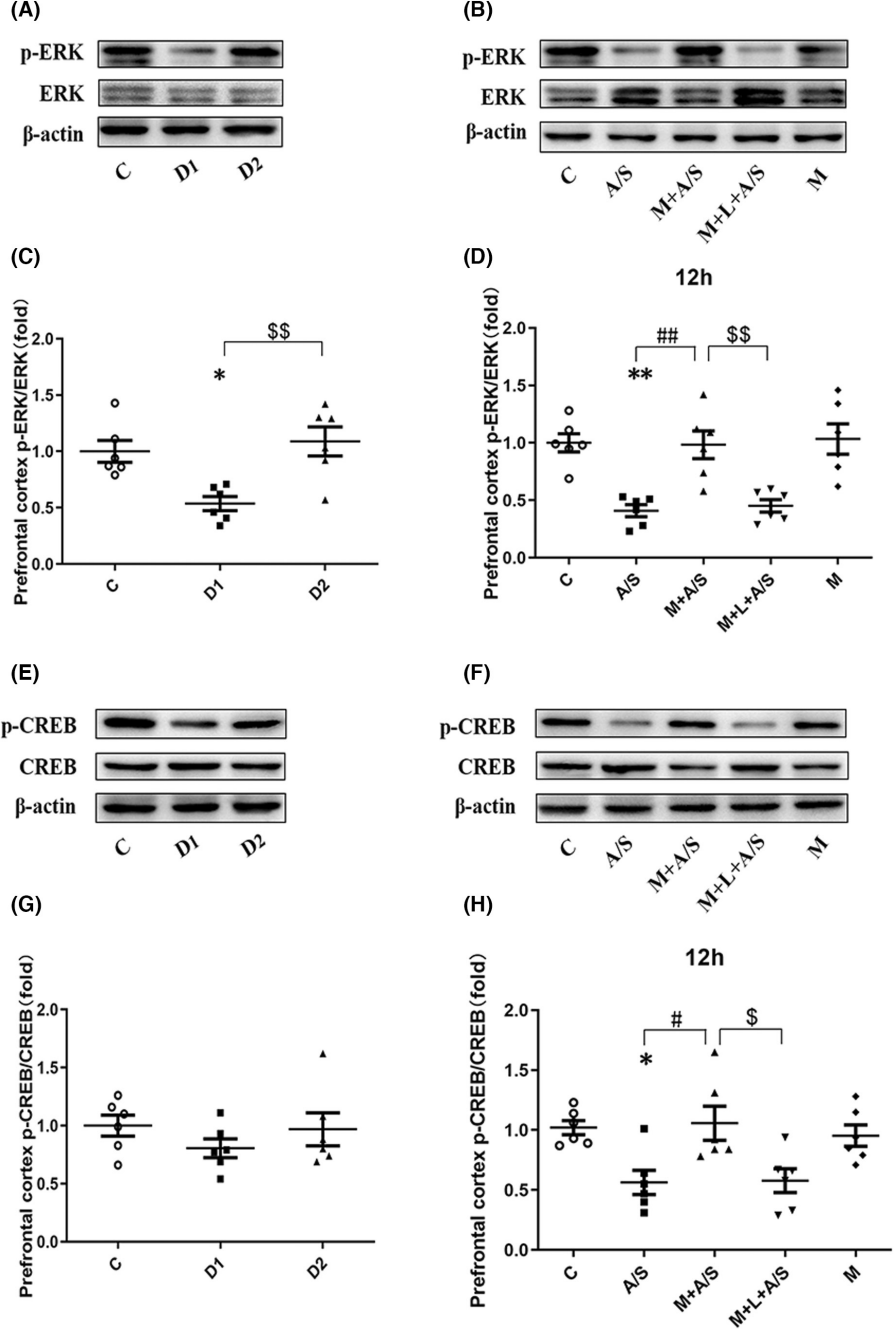
The ERK/CREB signal pathway in prefrontal cortex can be disturbed by laparotomy and preoperative administration of melatonin can improve the expression of ERK/CREB signal pathway related proteins. (A, C) The expression of p‐ERK/ERK was decreased 1 day after laparotomy (D1) and returned to normal on the second day (D2). (B, D) Melatonin pretreatment attenuated the decrease of p‐ERK/ERK expression 12 h after laparotomy and luzindole reverse the corrective action of melatonin. Melatonin alone did not change the p‐ERK/ERK expression. (E, G)The expression of p‐CREB/CREB was not decreased within 2 days after laparotomy (D1, D2). (F, H) Melatonin pretreatment attenuated the decrease of p‐CREB/CREB expression 12 h after laparotomy and luzindole reverse the corrective action of melatonin. Melatonin alone did not change the p‐CREB/CREB expression. *p < 0.05 and **p < 0.01, A/S group compared to C group; ^#^ *p* < 0.05 and ^##^ *p* < 0.01, M + A/S group compared to A/S group; ^$^ *p* < 0.05 and ^$$^ *p* < 0.01, M + L + A/S group compared to M + A/S group or the second day in A/S group compared to the first day after laparotomy; Data are presented as the mean ± SEM and analyzed by one‐way ANOVA followed by the Bonferroni post hoc analysis.

In the prefrontal cortex, p‐ERK/ERK was decreased on D1 after laparotomy (*p* < 0.05) and returned to normal on D2 (Figure 8A,C). When melatonin pretreatment was applied, the decrease in p‐ERK/ERK 12 h after laparotomy was alleviated, however, this improvement was antagonized by luzindole (all *p* < 0.05, Figure 8B,D). Compared with the control group, no significant difference in p‐CREB/CREB was observed on D1 and D2 after laparotomy (Figure 8E,G). When melatonin pretreatment was applied, the decrease in p‐CREB/CREB 12 h after laparotomy was alleviated, and this improvement was antagonized by luzindole (all *p* < 0.05). Likewise, melatonin alone did not trigger ERK and CREB phosphorylation in the prefrontal cortex (Figure 8F,H).

## 4 DISCUSSION

Our study showed that laparotomy under sevoflurane anesthesia had a greater influence than sevoflurane alone, leading to sleep disorders, sleep–wake rhythm shifts and delirium‐like behavior. Bmal1, Clock, and Cry1 mRNA expression showed peak shifts, MT_1_ receptor expression levels increased in the SCN, and p‐ERK/ERK and p‐CREB/CREB levels decreased in the hippocampus and prefrontal cortex of aged mice on D1 after laparotomy. Melatonin showed significant efficacy in ameliorating PSD and delirium‐like behavior, restoring the circadian rhythm, and resolving melatonin receptor and ERK/CREB pathway‐related protein expression abnormalities. In addition, most of the beneficial effect of melatonin was antagonized by melatonin receptor antagonist.

Studies^11^ in aged humans and animals indicate that circadian rhythms are not consistent throughout the life span. Both the rhythm amplitude and regularity deteriorate with age, and aging makes rhythms more vulnerable to changes in the external environment.^35^ A meta‐analysis by Lu et al.^16^ revealed that sleep and circadian interventions were associated with decreased incidences of POD. These findings prompted us to explore the role of circadian rhythms in POD in aged individuals and whether correcting dysrhythmia by melatonin pretreatment would improve POD.

To date, no animal model of delirium has been developed to accurately represent human delirium. Therefore, Peng et al.^32^ established an animal behavioral test battery to capture certain characteristics of POD, incorporating natural behavior (buried food and open field tests) to assess attention and awareness as well as learned behavior (Y maze test) to assess cognition. The present study using this model showed that laparotomy under anesthesia resulted in delirium‐like behavior in aged mice, while sevoflurane anesthesia alone for 2 h did not produce this effect.

The results were consistent with those of two previous studies using the same behavioral test battery^27^, ^36^ but isoflurane was used in those studies, and the effect of volatile anesthetics was not assessed separately. Sevoflurane was used in our study, which showed that sevoflurane anesthesia alone did not cause delirium‐like behavior in aged mice, demonstrating that laparotomy but not sevoflurane contributes to POD‐like behavior.

When observing sleep in mice, we found that sevoflurane alone and laparotomy under anesthesia both affected postoperative sleep. Of the two procedures, laparotomy under anesthesia had a greater impact, resulting in more sleep in the active phase, less sleep in the inactive phase, and greater changes in sleep episode duration, as well as a shift in the sleep–wake rhythm. The results indicated that laparotomy produced a more profound effect on postoperative sleep.

Although some studies, including one of our own, have reported that anesthesia or trauma may affect the circadian rhythm, the direction of the rhythm phase shift differed.^37^, ^38^, ^39^ A study by Song et al.^17^ found that after 5 h of isoflurane anesthesia, aged mice exhibited approximately 3 h of gross motor activity and acrophase delay, and Cry1, Per2, Bmal1, and Clock mRNA expression in the SCN displayed a peak delay or advance in young mice.

Our study is the first to investigate the changes in sleep–wake rhythms in aged mice using polygraphic recordings and vigilance state analysis. The sleep–wake rhythm shifted forward by approximately 2 h on D1 after laparotomy, and this shift disappeared on D2. Within the brain, the SCN of the mammalian hypothalamus is believed to be the primary regulator of circadian rhythms. We focused on Cry1, Per1, Bmal1, and Clock (clock genes) mRNA expression in the SCN and found that the peak delay (Cry1, Bmal1, and Clock) occurred in aged mice on D1 after laparotomy. The differences in rhythm phase shift directions reported in other studies may be caused by differences in intervention methods, intervention times, and experimental animals. It is noteworthy that our study found that melatonin could attenuate the behavior and circadian rhythm changes and correct phase shifts of clock gene expression in the SCN of aged mice induced by anesthesia or surgery. We also found that luzindole (melatonin antagonist) can antagonize these effects, indicating that melatonin receptors might be involved, at least partially, in PSD and delirium‐like behavior.

The MT_1_ and MT_2_ receptor distributions in the mouse brain are strikingly different. Compared with the MT_2_ subtype, the MT_1_ subtype exhibited greater expression in the SCN.^14^ In a post‐mortem study, Wu et al.^23^ found that the number of MT_1_‐ but not of MT_2_‐immunoreactive cells was increased in the SCN of depressed patients. Several studies^22^, ^40^ indicated that MT_1_, rather than MT_2_ receptors are involved in circadian rhythm regulation, and melatonin may regulate SCN function via MT_1_ receptors, with a minimal role of MT_2_ receptors.^41^ In our study, the differences in MT_1_ receptors among groups were consistent with clock gene levels in the SCN, suggesting that melatonin receptor disruption may cause circadian rhythm disorder after laparotomy under anesthesia, and application of melatonin to correct melatonin receptor disorders may improve this disorder. MT_2_ receptor expression in the SCN was not detected by western blot analysis, which is consistent with previous studies showing low‐MT_2_ receptor expression in the mouse SCN.^14^, ^41^

The hippocampus contributes to emotions, learning, and memory formation,^42^, ^43^, ^44^ and the prefrontal cortex is involved in encoding task‐relevant information in working memory.^45^ Moreover, the interaction between the hippocampus and prefrontal cortex plays an important role in attention and memory formation, consolidation, and expression.^46^, ^47^ Several studies^26^, ^48^, ^49^ have shown that the master circadian clock in the SCN is involved in neural function regulation in the hippocampus and prefrontal cortex. The ERK/CREB signaling pathway was reported to play a central role in the regulation of emotions, learning, and memory formation.^24^, ^50^, ^51^ A review by Snider^52^ reported that dysregulation of circadian timing affects learning and memory, which are hippocampal‐dependent tasks, and intracellular ERK signaling pathways were involved in this process.

Our findings suggest that POD‐like behavior is associated with decreased p‐ERK/ERK and p‐CREB/CREB levels in the hippocampus and prefrontal cortex, and these changes disappeared 1 day after surgery, which is consistent with the trend and time window of clock gene and melatonin receptor changes in the SCN. The M + A/S and M + L + A/S group results suggest that the ERK/CREB signaling pathway in the hippocampus and prefrontal cortex was affected by circadian rhythm changes, which may induced by melatonin receptors in the SCN.

Our study also found that melatonin can improve PSD and reverse sleep–wake rhythm changes, while melatonin antagonists can block the effect of melatonin; however, melatonin did not improve sleep in mice that had not undergone surgery. Studies have suggested that sleep disorders could be associated with disruption of normal circadian rhythms.^53^, ^54^ Currently, the efficacy of melatonin in the treatment of sleep disorders in the elderly is controversial,^55^ which may be because of the different administration routes and dosages of melatonin.^21^ However, meaningful effects of melatonin treatment have been reported in disorders associated with diminished or misaligned melatonin rhythms, such as circadian rhythm‐related sleep disorders, jet lag, shift work, and Alzheimer's disease.^21^ Additionally, MT_1_/MT_2_ receptors may play different roles in the 24‐h vigilance state.^56^ Our study also suggested that sleep disorders and sleep–wake rhythm changes in aged mice may be related to circadian rhythm disorders controlled by the SCN.

The present study has several limitations. First, the C57BL/6J mouse is a melatonin‐deficient mouse strain that we used to study the effect of exogenous melatonin on postoperative circadian rhythm disorders, and the relationship between light and the sleep–wake rhythm is different in humans and mice. Therefore, our results may not be directly applicable to humans. Second, we did not apply MT_1_ or MT_2_ receptor antagonists to more clearly distinguish the role of different melatonin receptors, which will be performed in future studies. Third, the mechanism of the postoperative circadian rhythm disorder affecting sleep structure is not discussed in this article, and this requires further study.

## 5 CONCLUSION

Laparotomy under sevoflurane anesthesia leads to sleep disorders and neurobehavioral changes in aged mice. Preoperative administration of melatonin can ameliorate PSDs and neurobehavioral abnormalities, and the mechanism may be related to alleviating abnormal melatonin receptor and clock gene expression in the SCN and correction of circadian rhythm disorders. The ERK/CREB signaling pathway in the hippocampus and prefrontal cortex may be involved in this process.

## AUTHOR CONTRIBUTIONS

XJ did conception and design, acquisition of data, interpretation of data, drafted and revised the manuscript. YS did drafted and revised the manuscript. YL did conception and design, drafted, and revised the manuscript. XG and JS were involved in acquisition of data, revising it critically for important intellectual content. Other authors were research consultant to XJ.

## FUNDING INFORMATION

This work was supported by the National Natural Science Foundation of China (Grant no. 81701052, to YL). The preparation of the manuscript was also supported in part by the National Institute on Drug Dependence and Beijing Key Laboratory of Drug Dependence.

## CONFLICT OF INTEREST STATEMENT

The authors declare that they have no competing interests.

## Data Availability

The datasets generated and/or analyzed during the current study are not publicly available but are available from the corresponding author on reasonable request.

## References

[1] Witlox J , Eurelings LS , de Jonghe JF , et al. Delirium in elderly patients and the risk of postdischarge mortality, institutionalization, and dementia: a meta‐analysis. JAMA. 2010;304(4):443‐451. 20664045 10.1001/jama.2010.1013

[2] Pisani MA , D'Ambrosio C . Sleep and delirium in adults who are critically ill: a contemporary review. Chest. 2020;157(4):977‐984. 31874132 10.1016/j.chest.2019.12.003

[3] Su X , Wang DX . Improve postoperative sleep: what can we do? Curr Opin Anaesthesiol. 2018;31(1):83‐88. 29120927 10.1097/ACO.0000000000000538 PMC5768217

[4] Su X , Meng ZT , Wu XH , et al. Dexmedetomidine for prevention of delirium in elderly patients after non‐cardiac surgery: a randomised, double‐blind, placebo‐controlled trial. Lancet. 2016;388(10054):1893‐1902. 27542303 10.1016/S0140-6736(16)30580-3

[5] Fong TG , Davis D , Growdon ME , Albuquerque A , Inouye SK . The interface between delirium and dementia in elderly adults. Lancet Neurol. 2015;14(8):823‐832. 26139023 10.1016/S1474-4422(15)00101-5 PMC4535349

[6] Kim SH , Kim N , Min KT , Kim EH , Oh H , Choi SH . Sleep disturbance and delirium in patients with acromegaly in the early postoperative period after transsphenoidal pituitary surgery. Medicine (Baltimore). 2020;99(45):e23157. 33158000 10.1097/MD.0000000000023157 PMC7647521

[7] Beydon L , Rauss A , Lofaso F , et al. Survey of the quality of sleep during the perioperative period. Study of factors predisposing to insomnia. Ann Fr Anesth Reanim. 1994;13(5):669‐674. 7733516 10.1016/s0750-7658(05)80723-3

[8] Kain ZN , Caldwell‐Andrews AA . Sleeping characteristics of adults undergoing outpatient elective surgery: a cohort study. J Clin Anesth. 2003;15(7):505‐509. 14698361 10.1016/j.jclinane.2003.02.002

[9] Dzierzewski JM , Dautovich N , Ravyts S . Sleep and cognition in older adults. Sleep Med Clin. 2018;13(1):93‐106. 29412987 10.1016/j.jsmc.2017.09.009 PMC5841581

[10] Berger M , Terrando N , Smith SK , Browndyke JN , Newman MF , Mathew JP . Neurocognitive function after cardiac surgery: from phenotypes to mechanisms. Anesthesiology. 2018;129(4):829‐851. 29621031 10.1097/ALN.0000000000002194 PMC6148379

[11] Hodges EL , Ashpole NM . Aging circadian rhythms and cannabinoids. Neurobiol Aging. 2019;79:110‐118. 31035036 10.1016/j.neurobiolaging.2019.03.008 PMC6591053

[12] Helfrich RF , Mander BA , Jagust WJ , Knight RT , Walker MP . Old brains come uncoupled in sleep: slow wave‐spindle synchrony, brain atrophy, and forgetting. Neuron. 2018;97(1):221‐230.e4. 29249289 10.1016/j.neuron.2017.11.020 PMC5754239

[13] Bellanti F , Iannelli G , Blonda M , et al. Alterations of clock gene RNA expression in brain regions of a triple transgenic model of Alzheimer's disease. J Alzheimers Dis. 2017;59(2):615‐631. 28671110 10.3233/JAD-160942 PMC5523844

[14] Klosen P , Lapmanee S , Schuster C , et al. MT_1_ and MT_2_ melatonin receptors are expressed in nonoverlapping neuronal populations. J Pineal Res. 2019;67(1):e12575. 30937953 10.1111/jpi.12575

[15] Karageorgiou E , Vossel KA . Brain rhythm attractor breakdown in Alzheimer's disease: functional and pathologic implications. Alzheimers Dement. 2017;13(9):1054‐1067. 28302453 10.1016/j.jalz.2017.02.003 PMC5585024

[16] Lu Y , Li YW , Wang L , et al. Promoting sleep and circadian health may prevent postoperative delirium: a systematic review and meta‐analysis of randomized clinical trials. Sleep Med Rev. 2019;48:101207. 31505369 10.1016/j.smrv.2019.08.001

[17] Song J , Chu S , Cui Y , et al. Circadian rhythm resynchronization improved isoflurane‐induced cognitive dysfunction in aged mice. Exp Neurol. 2018;306:45‐54. 29660304 10.1016/j.expneurol.2018.04.009

[18] Gandhi AV , Mosser EA , Oikonomou G , Prober DA . Melatonin is required for the circadian regulation of sleep. Neuron. 2015;85(6):1193‐1199. 25754820 10.1016/j.neuron.2015.02.016 PMC4851458

[19] Zhang Q , Gao F , Zhang S , Sun W , Li Z . Prophylactic use of exogenous melatonin and melatonin receptor agonists to improve sleep and delirium in the intensive care units: a systematic review and meta‐analysis of randomized controlled trials. Sleep Breath. 2019;23(4):1059‐1070. 31119597 10.1007/s11325-019-01831-5

[20] Liu Y , Ni C , Li Z , et al. Prophylactic melatonin attenuates isoflurane‐induced cognitive impairment in aged rats through hippocampal melatonin receptor 2–cAMP response element binding signalling. Basic Clin Pharmacol Toxicol. 2017;120(3):219‐226. 27515785 10.1111/bcpt.12652

[21] Zisapel N . New perspectives on the role of melatonin in human sleep, circadian rhythms and their regulation. Br J Pharmacol. 2018;175(16):3190‐3199. 29318587 10.1111/bph.14116 PMC6057895

[22] Comai S , Lopez‐Canul M , De Gregorio D , et al. Melatonin MT_1_ receptor as a novel target in neuropsychopharmacology: MT_1_ ligands, pathophysiological and therapeutic implications, and perspectives. Pharmacol Res. 2019;144:343‐356. 31029764 10.1016/j.phrs.2019.04.015

[23] Wu YH , Ursinus J , Zhou JN , et al. Alterations of melatonin receptors MT_1_ and MT_2_ in the hypothalamic suprachiasmatic nucleus during depression. J Affect Disord. 2013;148(2–3):357‐367. 23357659 10.1016/j.jad.2012.12.025

[24] Wang J , Jia Y , Li G , et al. The dopamine receptor D3 regulates lipopolysaccharide‐induced depressive‐like behavior in mice. Int J Neuropsychopharmacol. 2018;21(5):448‐460. 29390063 10.1093/ijnp/pyy005 PMC5932470

[25] Mikhail C , Vaucher A , Jimenez S , Tafti M . ERK signaling pathway regulates sleep duration through activity induced gene expression during wakefulness. Sci Signal. 2017;10(463):eaai9219. 28119463 10.1126/scisignal.aai9219

[26] Phan TX , Chan GC , Sindreu CB , et al. The diurnal oscillation of MAP (mitogen‐activated protein) kinase and adenylyl cyclase activities in the hippocampus depends on the suprachiasmatic nucleus. J Neurosci. 2011;31(29):10640‐10647. 21775607 10.1523/JNEUROSCI.6535-10.2011 PMC3146036

[27] Liufu N , Liu L , Shen S , et al. Anesthesia and surgery induce age‐dependent changes in behaviors and microbiota. Aging (Albany NY). 2020;12(2):1965‐1986. 31974315 10.18632/aging.102736 PMC7053599

[28] Lu Y , Chen L , Ye J , et al. Surgery/anesthesia disturbs mitochondrial fission/fusion dynamics in the brain of aged mice with postoperative delirium. Aging (Albany NY). 2020;12(1):844‐865. 31929114 10.18632/aging.102659 PMC6977661

[29] Guo P , Pi H , Xu S , et al. Melatonin improves mitochondrial function by promoting MT_1_/SIRT_1_/PGC‐1 alpha‐dependent mitochondrial biogenesis in cadmium‐induced hepatotoxicity in vitro. Toxicol Sci. 2014;142(1):182‐195. 25159133 10.1093/toxsci/kfu164 PMC4226765

[30] Wang H , Ma R , Fang H , Xue ZG , Liao QW . Impaired spatial learning memory after isoflurane anesthesia or appendectomy in aged mice is associated with microglia activation. J Cell Death. 2015;8:9‐19. 26380557 10.4137/JCD.S30596 PMC4560456

[31] Li Y , Pan K , Chen L , et al. Deferoxamine regulates neuroinflammation and iron homeostasis in a mouse model of postoperative cognitive dysfunction. J Neuroinflammation. 2016;13(1):268. 27733186 10.1186/s12974-016-0740-2 PMC5062909

[32] Peng M , Zhang C , Dong Y , et al. Battery of behavioral tests in mice to study postoperative delirium. Sci Rep. 2016;6:29874. 27435513 10.1038/srep29874 PMC4951688

[33] Oishi Y , Takata Y , Taguchi Y , Kohtoh S , Urade Y , Lazarus M . Polygraphic recording procedure for measuring sleep in mice. J Vis Exp. 2016;107:e53678. 10.3791/53678 PMC4781694 26863349

[34] Chen CR , Zhou XZ , Luo YJ , Huang ZL , Urade Y , Qu WM . Magnolol, a major bioactive constituent of the bark of *Magnolia officinalis*, induces sleep via the benzodiazepine site of GABA(a) receptor in mice. Neuropharmacology. 2012;63(6):1191‐1199. 22771461 10.1016/j.neuropharm.2012.06.031

[35] Nakamura TJ , Takasu NN , Nakamura W . The suprachiasmatic nucleus: age‐related decline in biological rhythms. J Physiol Sci. 2016;66(5):367‐374. 26915078 10.1007/s12576-016-0439-2 PMC10717791

[36] Liu T , Li Z , He J , et al. Regional metabolic patterns of abnormal postoperative behavioral performance in aged mice assessed by ^1^H‐NMR dynamic mapping method. Neurosci Bull. 2020;36(1):25‐38. 31376056 10.1007/s12264-019-00414-4 PMC6940420

[37] Coiffard B , Diallo AB , Culver A , et al. Circadian rhythm disruption and sepsis in severe trauma patients. Shock. 2019;52(1):29‐36. 30074979 10.1097/SHK.0000000000001241

[38] Poulsen RC , Warman GR , Sleigh J , Ludin NM , Cheeseman JF . How does general anaesthesia affect the circadian clock? Sleep Med Rev. 2018;37:35‐44. 28162920 10.1016/j.smrv.2016.12.002

[39] Song Y , Liu Y , Yuan Y , et al. Effects of general versus subarachnoid anaesthesia on circadian melatonin rhythm and postoperative delirium in elderly patients undergoing hip fracture surgery: a prospective cohort clinical trial. EBioMedicine. 2021;70:103490. 34280784 10.1016/j.ebiom.2021.103490 PMC8318871

[40] Stein RM , Kang HJ , McCorvy JD , et al. Virtual discovery of melatonin receptor ligands to modulate circadian rhythms. Nature. 2020;579(7800):609‐614. 32040955 10.1038/s41586-020-2027-0 PMC7134359

[41] Waly NE , Hallworth R . Circadian pattern of melatonin MT_1_ and MT_2_ receptor localization in the rat suprachiasmatic nucleus. J Circadian Rhythms. 2015;13:1. 27103927 10.5334/jcr.ab PMC4831275

[42] Jimenez JC , Su K , Goldberg AR , et al. Anxiety cells in a hippocampal‐hypothalamic circuit. Neuron. 2018;97(3):670‐683.e6. 29397273 10.1016/j.neuron.2018.01.016 PMC5877404

[43] Tyng CM , Amin HU , Saad MNM , Malik AS . The influences of emotion on learning and memory. Front Psychol. 2017;8:1454. 28883804 10.3389/fpsyg.2017.01454 PMC5573739

[44] Lisman J , Buzsaki G , Eichenbaum H , et al. Viewpoints: how the hippocampus contributes to memory, navigation and cognition. Nat Neurosci. 2017;20(11):1434‐1447. 29073641 10.1038/nn.4661 PMC5943637

[45] Lara AH , Wallis JD . The role of prefrontal cortex in working memory: a mini review. Front Syst Neurosci. 2015;9:173. 26733825 10.3389/fnsys.2015.00173 PMC4683174

[46] Tan Z , Robinson HL , Yin DM , et al. Dynamic ErbB4 activity in hippocampal‐prefrontal synchrony and top‐down attention in rodents. Neuron. 2018;98(2):380‐393.e4. 29628188 10.1016/j.neuron.2018.03.018 PMC5909841

[47] Maingret N , Girardeau G , Todorova R , Goutierre M , Zugaro M . Hippocampo‐cortical coupling mediates memory consolidation during sleep. Nat Neurosci. 2016;19(7):959‐964. 27182818 10.1038/nn.4304

[48] Naseri Kouzehgarani G , Bothwell MY , Gillette MU . Circadian rhythm of redox state regulates membrane excitability in hippocampal CA1 neurons. Eur J Neurosci. 2020;51(1):34‐46. 30614107 10.1111/ejn.14334 PMC6609501

[49] Ikeno T , Yan L . Chronic light exposure in the middle of the night disturbs the circadian system and emotional regulation. J Biol Rhythms. 2016;31(4):352‐364. 27075857 10.1177/0748730416642065

[50] Zhao X , Li S , Gaur U , Zheng W . Artemisinin improved neuronal functions in Alzheimer's disease animal model 3xtg mice and neuronal cells via stimulating the ERK/CREB signaling pathway. Aging Dis. 2020;11(4):801‐819. 32765947 10.14336/AD.2019.0813 PMC7390534

[51] Ge JF , Xu YY , Qin G , Pan XY , Cheng JQ , Chen FH . Nesfatin‐1, a potent anorexic agent, decreases exploration and induces anxiety‐like behavior in rats without altering learning or memory. Brain Res. 2015;1629:171‐181. 26498879 10.1016/j.brainres.2015.10.027

[52] Snider KH , Sullivan KA , Obrietan K . Circadian regulation of hippocampal‐dependent memory: circuits, synapses, and molecular mechanisms. Neural Plast. 2018;2018:7292540. 29593785 10.1155/2018/7292540 PMC5822921

[53] Musiek ES , Holtzman DM . Mechanisms linking circadian clocks, sleep, and neurodegeneration. Science. 2016;354(6315):1004‐1008. 27885006 10.1126/science.aah4968 PMC5219881

[54] Weissova K , Skrabalova J , Skalova K , et al. Circadian rhythms of melatonin and peripheral clock gene expression in idiopathic REM sleep behavior disorder. Sleep Med. 2018;52:1‐6. 30195196 10.1016/j.sleep.2018.07.019

[55] Gobbi G , Comai S . Differential function of melatonin MT_1_ and MT_2_ receptors in REM and NREM sleep. Front Endocrinol (Lausanne). 2019;10:87. 30881340 10.3389/fendo.2019.00087 PMC6407453

[56] Ochoa‐Sanchez R , Comai S , Spadoni G , Bedini A , Tarzia G , Gobbi G . Melatonin, selective and non‐selective MT_1_/MT_2_ receptors agonists: differential effects on the 24‐h vigilance states. Neurosci Lett. 2014;561:156‐161. 24406151 10.1016/j.neulet.2013.12.069

